# From Macromolecule to Microbe: Identification of *Ligilactobacillus salivarius* D3-8 as a Key Degrader of Ejiao and a Novel Therapeutic Probiotic for Ulcerative Colitis

**DOI:** 10.3390/nu18060947

**Published:** 2026-03-17

**Authors:** Wei Dai, Mingfeng Ma, Qin Feng, Xiaobo Duan, Yaru Zhang, Xiaoying Zhou, Haibin Liu, Qingsen Shang

**Affiliations:** 1 Key Laboratory of Marine Drugs of Ministry of Education, Shandong Key Laboratory of Glycoscience and Glycotherapeutics, School of Medicine and Pharmacy, Ocean University of China, Qingdao 266003, China; daiwei3266@stu.ouc.edu.cn (W.D.); mmf621121@163.com (M.M.); 2 Laboratory for Marine Drugs and Bioproducts, Qingdao Marine Science and Technology Center, Qingdao 266237, China; 3 Marine Biomedical Research Institute of Qingdao, Qingdao 266071, China; 4 Shandong Key Laboratory of Gelatine Medicines Research and Development, Dong’e Ejiao Co., Ltd., Liaocheng 252201, China; fengqin39@dongeejiao.com (Q.F.); duanxb@dongeejiao.com (X.D.); zhangyaru@dongeejiao.com (Y.Z.); zhouxiaoying50@dongeejiao.com (X.Z.)

**Keywords:** Ejiao, gut microbiota, ulcerative colitis, macromolecular protein, fermentation, *Ligilactobacillus salivarius*, peptide, indole-3-carbinol, degradation, dextran sodium sulfate

## Abstract

**Background/Objectives**: Ejiao, a macromolecular protein complex derived from donkey hide, is a traditional Chinese medicine with clinically demonstrated efficacy against ulcerative colitis (UC). Due to its large molecular size and poor absorbability, its therapeutic effects are presumed to depend on gut microbiota. We hypothesized that specific gut bacteria capable of degrading Ejiao might also mediate its biological functions. **Methods**: To test this hypothesis, a systematic investigation was conducted by integrating culturomics, proteomics, metabolomics, 16S rRNA gene amplicon high-throughput sequencing, and animal disease models. **Results**: A total of 134 human gut bacterial strains capable of utilizing Ejiao as a nutrient source were isolated. Among them, *Ligilactobacillus salivarius* D3-8 exhibited the strongest growth in Ejiao-based medium. Genomic analysis identified 63 protease/peptidase genes, and peptidomic profiling confirmed its degradation activity, which released 50 novel peptides. Notably, administration of *L. salivarius* D3-8 alone significantly alleviated dextran sodium sulfate (DSS)-induced colitis, concurrently increasing the abundance of beneficial bacterium *Dubosiella newyorkensis* and elevating the anti-inflammatory metabolite indole-3-carbinol via upregulated tryptophan metabolism. **Conclusions**: Our findings identify *L. salivarius* D3-8 as both a dedicated Ejiao-degrader and a protective probiotic against colitis. This work provides direct evidence that gut bacteria can utilize Ejiao and proposes a potential novel mechanistic framework in which the biological effects of Ejiao may be mediated through its interaction with specific, functionally potent degraders such as *L. salivarius* D3-8.

## 1. Introduction

Ulcerative colitis (UC), a chronic and relapsing inflammatory bowel disease (IBD), poses a significant challenge to global health due to its complex etiology and limited long-term therapeutic options [1,2,3]. In this context, traditional Chinese medicines (TCMs) have garnered increasing attention as potential complementary or alternative therapies [4,5,6]. Ejiao (*Asini Corii Colla*), a gelatinous preparation derived from donkey (*Equus asinus* L.) hide, is a renowned TCM and tonic food historically used for nourishing the blood and improving hematopoiesis [7,8,9]. Recently, both animal studies and clinical trials have demonstrated that Ejiao can alleviate symptoms of UC [10,11]. However, a fundamental paradox surrounds its mechanism of action.

Unlike many small-molecule drugs or dietary nutrients, Ejiao is not a single, well-defined compound but a macromolecular protein complex with high molecular weight [7,12]. The macromolecular nature of Ejiao results in poor intestinal absorption of its intact form, making it unlikely to exert direct pharmacological or nutritional effects on host cells through conventional pathways [7,12]. Consequently, the classical “drug–receptor” interaction model is not sufficient to explain its bioactivity, shifting scientific inquiry toward the gut luminal environment [13,14].

The gut microbiota, a vast and complex ecosystem, is increasingly recognized as a crucial mediator of the bioavailability and efficacy of orally administered TCMs, particularly for macromolecular and poorly absorbable constituents [15,16,17]. It is hypothesized that the gut bacteria can metabolize these complex compounds into smaller, absorbable, and biologically active metabolites [15,18]. For Ejiao, its therapeutic effects against UC are therefore presumed to depend on the gut microbiota [11,13].

However, although correlative studies have observed shifts in microbial communities following Ejiao administration, the field still faces a critical knowledge gap. The existing evidence remains largely associative, failing to move beyond correlation to establish direct causation [11,13]. A pivotal, unanswered question is: which specific bacterial taxa are capable of directly interacting with and biotransforming the Ejiao macromolecule, and are these degraders merely passive utilizers or active mediators of its therapeutic functions? Identifying these key microbial players and elucidating their functional roles is essential to move from a black-box understanding to a mechanistic explanation of Ejiao’s action.

To bridge this gap, we posited a targeted hypothesis: specific gut bacteria capable of degrading Ejiao are not just consumers of this nutrient source but are direct executors or key intermediates in conveying its anti-colitis effects. Testing this hypothesis requires a departure from purely observational microbiome profiling and the adoption of a directed culturomics and functional validation approach. In this study, we therefore aimed to: (1) isolate and screen human gut bacteria that can utilize Ejiao as a sole nutrient source; (2) from these, identify and characterize a prime degrader strain with potent activity; and (3) evaluate whether this specific bacterium alone can recapitulate or mediate the protective effects of Ejiao in a murine model of colitis, and if so, uncover the underlying mechanisms.

## 2. Materials and Methods

### 2.1. Chemicals and Reagents

Ejiao was provided by Dong’e Ejiao Co., Ltd. (Liaocheng, Shandong, China). Agar was sourced from Sangon Biotech (Shanghai, China) and used for the preparation of solid culture media. MRS medium was acquired from Hope Bio-Technology Co., Ltd. (Qingdao, Shandong, China). Dextran sodium sulfate (DSS) was obtained from MP Biomedicals (Solon, OH, USA). The standard solutions of short-chain fatty acids (SCFAs), including lactate, acetate, propionate, butyrate, valerate, lactate and succinate, were all purchased from Sigma-Aldrich (St. Louis, MO, USA).

### 2.2. Isolation of the Ejiao-Degrading Bacteria

Ten healthy volunteers (6 females and 4 males), aged 22–26 years, were recruited for this study. None of the participants had received antibiotics, probiotics, or prebiotics for at least six months prior to sample collection. The study protocol for fecal sample collection was reviewed and approved by the Ethical Committee of Qingdao Marine Biomedical Research Institute (Approval No. E-MBEJ-2024-02-26).

Liquid Ejiao medium was prepared by dissolving Ejiao in distilled water to a final concentration of 8 g/L. Ejiao was added to the medium as the sole nutrient source. Under these conditions, bacterial growth can only occur if the strain possesses the enzymatic capacity to degrade and metabolize Ejiao components. The liquid formulation was solidified with 1.2% (*w*/*v*) agar to prepare solid medium. Bacterial isolation and purification were performed as previously described [19,20]. Briefly, fecal samples were homogenized and resuspended in 0.1 M anaerobic phosphate-buffered saline (PBS, pH 7.0) to obtain 20% (*w*/*v*) suspensions. One milliliter of each suspension was inoculated into 50 mL of liquid Ejiao medium and incubated anaerobically at 37 °C for 48 h. After incubation, cultures were serially diluted (10-fold steps) and plated onto solid Ejiao medium. All procedures were carried out in an Electrotek AW 500SG (Shipley, West Yorkshire, UK) anaerobic workstation maintaining an atmosphere of 80% N_2_, 10% H_2_, and 10% CO_2_.

Single colonies were picked, inoculated into fresh liquid Ejiao medium, and grown for 48 h under the same anaerobic conditions. The 16S rRNA genes of the isolated bacteria were amplified using universal primers 27F and 1492R. The resulting PCR products were sequenced by Sangon Biotech (Shanghai, China). Taxonomic assignment was conducted using the EzBioCloud database (https://www.ezbiocloud.net/, accessed on 11 March 2025) following the established methodology [21]. A phylogenetic tree was constructed based on the 16S rDNA sequences of bacterial isolates using the Molecular Evolutionary Genetics Analysis (MEGA) software (version 7.0.26) as previously described [22].

A representative strain from each bacterial species was randomly selected and cultured in liquid Ejiao medium. As mentioned above, the medium contained Ejiao at a concentration of 8 g/L as the sole nutrient source. Bacterial growth was monitored by measuring the optical density at 600 nm (OD_600_) at designated time points using a ReadMax 1200 microplate spectrophotometer from Flash Spectrum Biological Technology (Shanghai, China). To compare degradative capacity among positive strains, growth curves were generated using OD_600_ values measured in Ejiao-supplemented medium; because all strains were cultured under identical conditions, differences in growth kinetics directly reflect differences in their ability to metabolize Ejiao. After cultivation, the concentrations of SCFAs in the culture supernatant were determined as previously described [19,20]. The fermentation experiments were conducted with four biological replicates.

### 2.3. Genomic Analysis of L. salivarius D3-8

For genomic analysis, *L. salivarius* D3-8 was first revived from −80 °C frozen stock in MRS broth. After 24 h of anaerobic incubation at 37 °C, the culture was inoculated at 1% (*v*/*v*) into 1 L of fresh anaerobic Ejiao medium and further fermented under the same conditions. Bacterial cells were harvested during the exponential phase by centrifugation at 12,000 rpm for 10 min. The pellet was submitted to Majorbio Bio-Pharm Biotechnology (Shanghai, China) for whole-genome sequencing using both the Illumina HiSeq platform and the Oxford Nanopore Technologies (ONT) Nanopore PromethION platform (Oxford, Cambridge, UK). Functional annotation based on Clusters of Orthologous Genes (COG) and other bioinformatic analyses were performed using the Majorbio Cloud Platform (www.majorbio.com, accessed on 30 June 2025) according to well-established protocols [23]. All fermentation steps were carried out anaerobically (80% N_2_, 10% H_2_ and 10% CO_2_) at 37 °C in an Electrotek AW 500SG anaerobic chamber (Shipley, West Yorkshire, UK).

### 2.4. Peptide Analysis of Ejiao Fermented by L. salivarius D3-8

*L. salivarius* D3-8 was cultivated in 1 L of anaerobic liquid Ejiao medium to investigate its proteolytic activity. To monitor the dynamic changes in the peptide profile, cell-free supernatants were aseptically collected at four key time points (0, 6, 24, and 72 h) post-inoculation. The samples were immediately centrifuged (12,000× *g*, 10 min, 4 °C) to remove bacterial cells. The resulting supernatants were then frozen at −80 °C and subsequently lyophilized to complete dryness for 48 h using a vacuum freeze-dryer from Bilon Instrument (Shanghai, China) to concentrate the peptides and prevent degradation. All lyophilized samples were shipped to Majorbio Bio-Pharm Biotechnology (Shanghai, China) for comprehensive peptidomic analysis.

At the service provider, peptides were extracted from the samples and analyzed using high-resolution liquid chromatography-tandem mass spectrometry (LC-MS/MS) as previously described [24]. An Orbitrap Astral mass spectrometer from Thermo Fisher Scientific (Waltham, MA, USA) was applied for the analysis. The LC separation was performed on an uPAC High Throughput column (75 μm × 5.5 cm) from Thermo Fisher Scientific (Waltham, MA, USA) using solvent A (water with 2% acetonitrile and 0.1% formic acid) and solvent B (water with 80% acetonitrile and 0.1% formic acid).

Peptide identification was performed using the PEAKS Studio software (version 8.5) from Bioinformatics Solutions Inc. (Waterloo, ON, Canada) against the *Equus asinus* (Donkey) database. To visualize and interpret the temporal changes in the peptidome, principal component analysis (PCA) and hierarchical clustering analysis were performed on the normalized peptide abundance data to systematically identify peptides that were significantly upregulated or downregulated across the four fermentation time points.

### 2.5. Animal Treatment and Sample Collection

All animal procedures were approved by the Ethics Committee of the Qingdao Marine Biomedical Research Institute (Approval No. E-MBJQ-2025-03-26). Fifty specific-pathogen-free (SPF) male C57BL/6J mice (7 weeks old) were purchased from Vital River Laboratory Animal Technology (Beijing, China) and housed under standard specific pathogen-free (SPF) conditions at a controlled temperature of 23 ± 1 °C with a 12-h light/dark cycle. After one week of acclimatization, the mice were randomly assigned to five groups (*n* = 10 per group):

(1) Normal control (NC): received regular drinking water throughout the experiment.
(2) Model (MD): received 2.2% (*w*/*v*) dextran sulfate sodium (DSS) in drinking water for the first 6 days.
(3) Low-dose *L. salivarius* D3-8 (LSL): DSS as in MD group, plus daily oral gavage of 3.14 × 10^7^ CFU/mouse.
(4) Medium-dose *L. salivarius* D3-8 (LSM): DSS as in MD group, plus daily oral gavage of 3.14 × 10^8^ CFU/mouse.
(5) High-dose *L. salivarius* D3-8 (LSH): DSS as in MD group, plus daily oral gavage of 3.14 × 10^9^ CFU/mouse.

To minimize pain and distress, all animal manipulations, including oral gavage, were performed gently and with minimal handling stress. Throughout the 8-day experimental period, the health status of all mice was monitored at least twice daily. Clinical signs assessed included body weight loss, stool consistency, rectal bleeding, piloerection, hunched posture, and general mobility. Humane endpoints were established a priori to prevent unnecessary suffering. Mice were to be immediately euthanized if they exhibited any of the following criteria: body weight loss exceeding 20% of their initial body weight, signs of severe morbidity, or the presence of rectal prolapse. During this study, no mice met these humane endpoint criteria and all animals survived until the scheduled termination point.

*L. salivarius* D3-8 cells were harvested by centrifugation (8000× *g*, 10 min), washed, and resuspended in sterile anaerobic PBS to the required concentrations. Bacterial suspensions or PBS vehicles (for NC and MD groups) were administered by oral gavage daily for 7 days, starting one day before DSS exposure. At the end of the experiment on day 8, all mice were humanely euthanized by cervical dislocation under deep anesthesia induced by intraperitoneal injection of pentobarbital sodium (50 mg/kg body weight), in accordance with the American Veterinary Medical Association (AVMA) Guidelines for the Euthanasia of Animals (https://www.avma.org/resources-tools/avma-policies/avma-guidelines-euthanasia-animals, accessed on 26 March 2025). Colon and cecal tissues were collected, measured for length, and immediately fixed in 4% paraformaldehyde for hematoxylin and eosin (H&E) staining. Histopathological scoring of colon sections was performed in a blinded manner according to established criteria [25].

### 2.6. 16S rRNA Gene Amplicon Sequencing and Bioinformatic Analyses

Total genomic DNA was extracted from fecal samples of mice using a SPINeasy DNA kit for feces from MP Biomedicals (Solon, OH, USA). DNA quality was assessed, and the V3–V4 hypervariable regions of the bacterial 16S rRNA gene were amplified with primers 338F (5′-ACTCCTACGGGAGGCAGCAG-3′) and 806R (5′-GGACTACHVGGGTWTCTAAT-3′). The amplicons were sequenced on an Illumina PE300 platform from Majorbio Bio-Pharm Biotechnology (Shanghai, China). Bioinformatic analyses, including PCA, heatmap visualization, and Wilcoxon rank-sum tests, were all performed using online tools from the Majorbio Cloud Platform (www.majorbio.com, accessed on 16 June 2025) as previously described [23].

### 2.7. Metabolomic Analysis

Fecal samples (approximately 60 mg each) were collected from six randomly selected mice per group at the endpoint. Residual samples from other mice within the same group were retained as backups to compensate for potential insufficient material during processing. For quality control (QC), an equal-volume aliquot from each sample was pooled to create a composite QC sample. Metabolomic profiling was performed using liquid chromatography–tandem mass spectrometry (LC-MS/MS) in both positive and negative electrospray ionization modes. Raw MS data were processed with Progenesis QI software (v2.0) from Waters Corporation (Milford, CT, USA) for peak picking, alignment, and normalization.

Metabolite identification was carried out by matching accurate mass and MS/MS spectra against the Human Metabolome Database (HMDB) (http://www.hmdb.ca/, accessed on 28 June 2025), Metlin (https://metlin.scripps.edu/, accessed on 28 June 2025), and the in-house Majorbio Database. All downstream statistical analyses, including non-metric multidimensional scaling (NMDS) analysis, variable importance in projection (VIP) analysis, and Kyoto Encyclopedia of Genes and Genomes (KEGG) pathway enrichment analysis, were performed using the online tools from the Majorbio cloud platform (https://cloud.majorbio.com, accessed on 30 June 2025) [23]. Significantly differential metabolites were selected with a threshold of VIP > 1 and *p* < 0.05 (Student’s *t*-test).

### 2.8. Statistical Analysis

All the statistical analyses were performed using GraphPad Prism (version 8.0.2) (San Diego, CA, USA). All data are presented as the mean ± standard error of the mean (SEM). For all experiments, sample sizes (*n*) are indicated in the figure legends and represent biologically independent animals or replicate cultures, as specified. Prior to analysis, the normality of data distribution was assessed using the Shapiro–Wilk test. Homogeneity of variances was evaluated using the Brown–Forsythe test. When both normality and homogeneity of variances assumptions were met, parametric tests were applied as described below. For comparisons between two groups, statistical significance was determined using the two-tailed Student’s *t*-test. For comparisons involving three or more groups, one-way or two-way analysis of variance (ANOVA) was performed, followed by Tukey’s honestly significant difference (HSD) post hoc test for multiple comparisons. For all analyses, differences were considered statistically significant at *p* < 0.05. Significance levels are denoted in the figures as follows: * *p* < 0.05 versus NC group; ** *p* < 0.01 versus NC group; # *p* < 0.05 versus MD group; ## *p* < 0.01 versus MD group.

## 3. Results

### 3.1. Isolation and Screening Identify L. salivarius D3-8 as a Key Ejiao-Degrading Bacterium in the Human Gut

**Figure 1:**
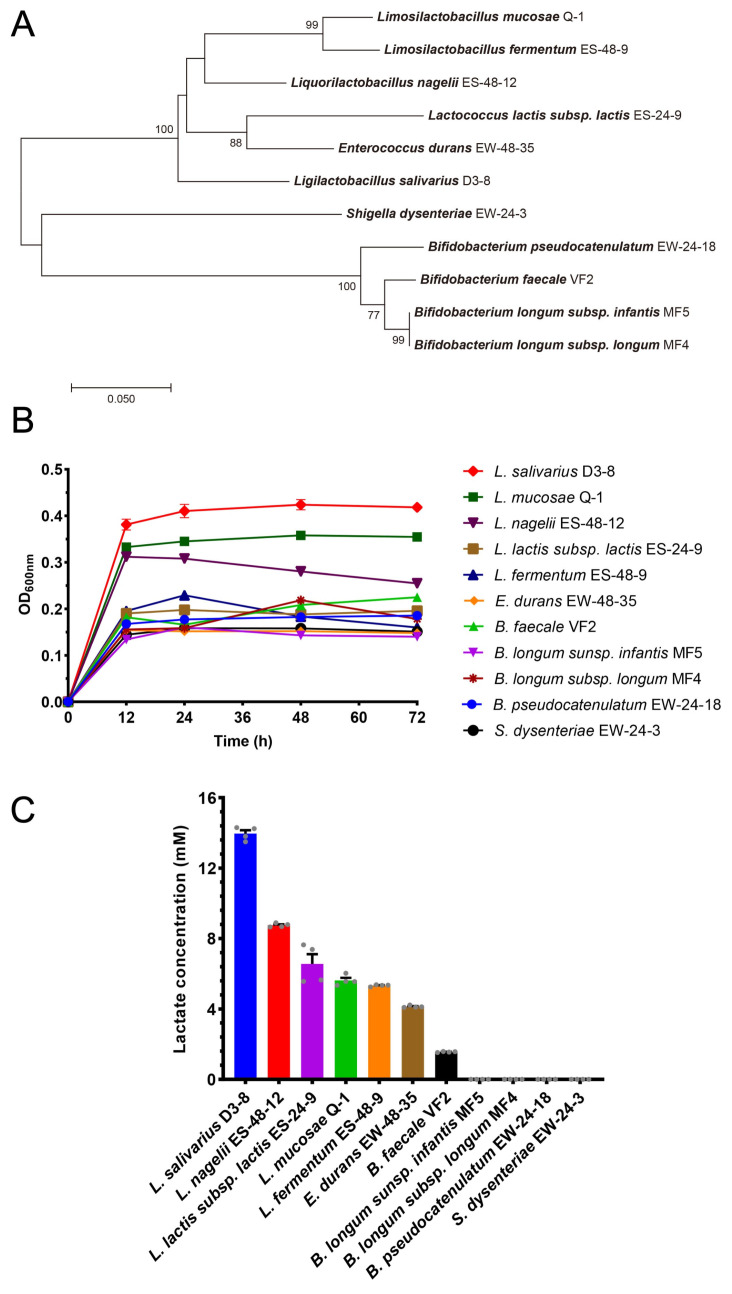
Screening and characterization of different bacterial strains for Ejiao fermentation. The fermentation experiments were conducted with four biological replicates (*n* = 4 per group). Phylogenetic tree of the isolated bacteria based on the 16S rRNA gene sequences (**A**). One representative strain from each species was selected for the analysis. Growth curves of each strain cultured in Ejiao-based medium (**B**). SCFA production profiles after fermentation in Ejiao medium (**C**). The grey dots in panel C represent the experimental values.

Fecal samples from ten healthy donors were cultured in Ejiao-containing medium, leading to the isolation of 134 bacterial strains belonging to 11 species across 8 genera (Table 1). One representative strain from each species was selected for subsequent characterization. Phylogenetic analysis based on 16S rRNA gene sequences positioned these 11 selected strains within their respective taxonomic groups (Figure 1A). Growth curve analysis revealed that *L. salivarius* D3-8 reached the highest optical density (OD_600_ ≈ 0.43) when cultivated in liquid Ejiao medium, outperforming all other tested strains (Figure 1B).

Given the crucial role of microbial-derived SCFAs in intestinal homeostasis [26,27], we next profiled the SCFA production by these Ejiao-degrading isolates. While lactate was the primary SCFAs detected, *L. salivarius* D3-8 produced it at the highest concentration under these conditions (Figure 1C). Collectively, based on its superior growth and lactate production in Ejiao-based culture, *L. salivarius* D3-8 was identified as a potent and promising keystone bacterium responsible for Ejiao degradation in the gut.

### 3.2. Genomic Analysis Reveals the Proteolytic Arsenal of the Keystone Bacterium L. salivarius D3-8

**Figure 2:**
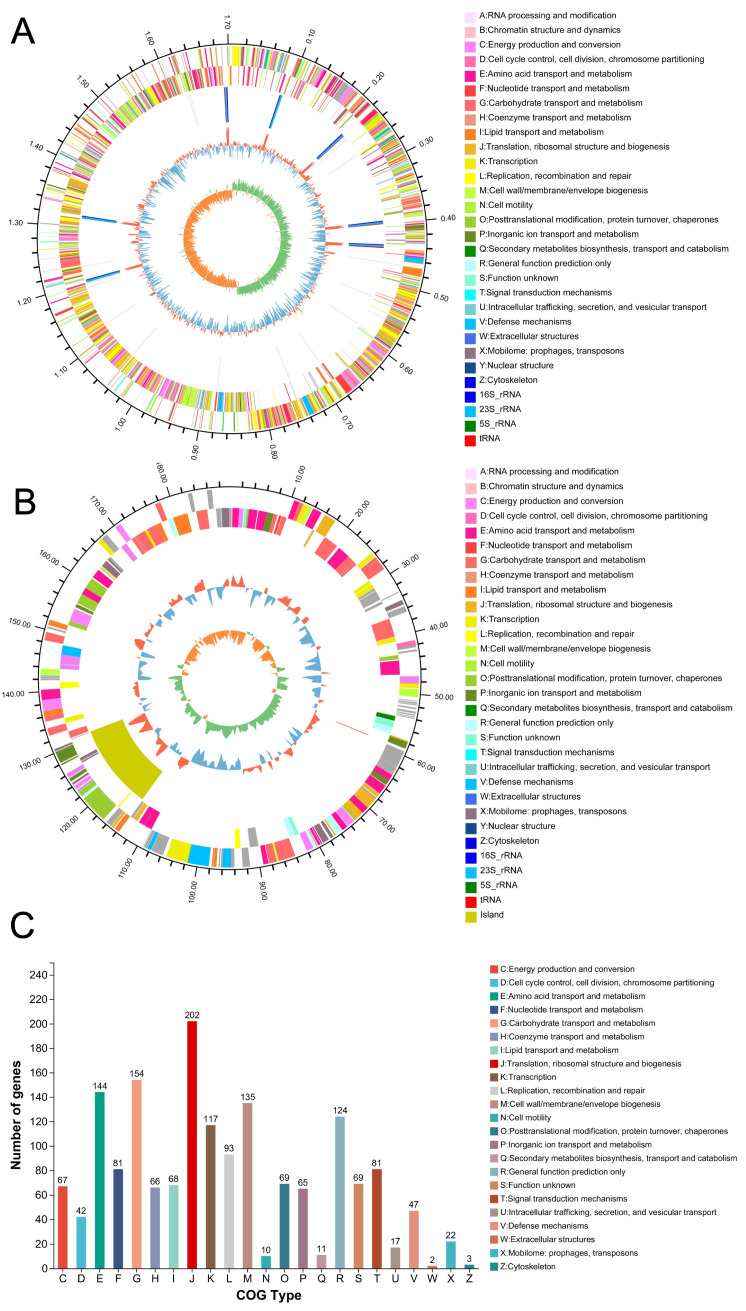
Genomic features and functional potential of *L. salivarius* D3-8. Circular map of the complete chromosome (**A**). Circular map of the plasmid (**B**). Functional classification of predicted genes based on COG analysis (**C**).

The robust growth of *L. salivarius* D3-8 in Ejiao medium confirmed its capacity to utilize this substrate, prompting us to sequence its genome to elucidate the underlying degradation machinery. The complete genome comprises one chromosome (1,705,526 bp) and one plasmid (189,583 bp), with an overall G+C content of 32.99% (Figure 2A,B). Given that many health-beneficial bioactive peptides are derived from collagen and that lactic acid bacteria are known producers of such peptides due to their proteolytic systems [28,29,30], we next focused our annotation on protease-related genes.

Clusters of Orthologous Groups (COG) functional analysis identified a substantial repertoire of proteolytic enzymes within the genome of *L. salivarius* D3-8, including 25 genes encoding proteases and 38 genes encoding peptidases (Figure 2C, Table 2 and Table 3). This genetic endowment strongly supports our hypothesis that *L. salivarius* D3-8 can degrade type I collagen-rich Ejiao into bioactive peptides.

### 3.3. Peptidomic Profiling Validates Ejiao Degradation and Identifies Novel Bioactive Peptides

**Figure 3:**
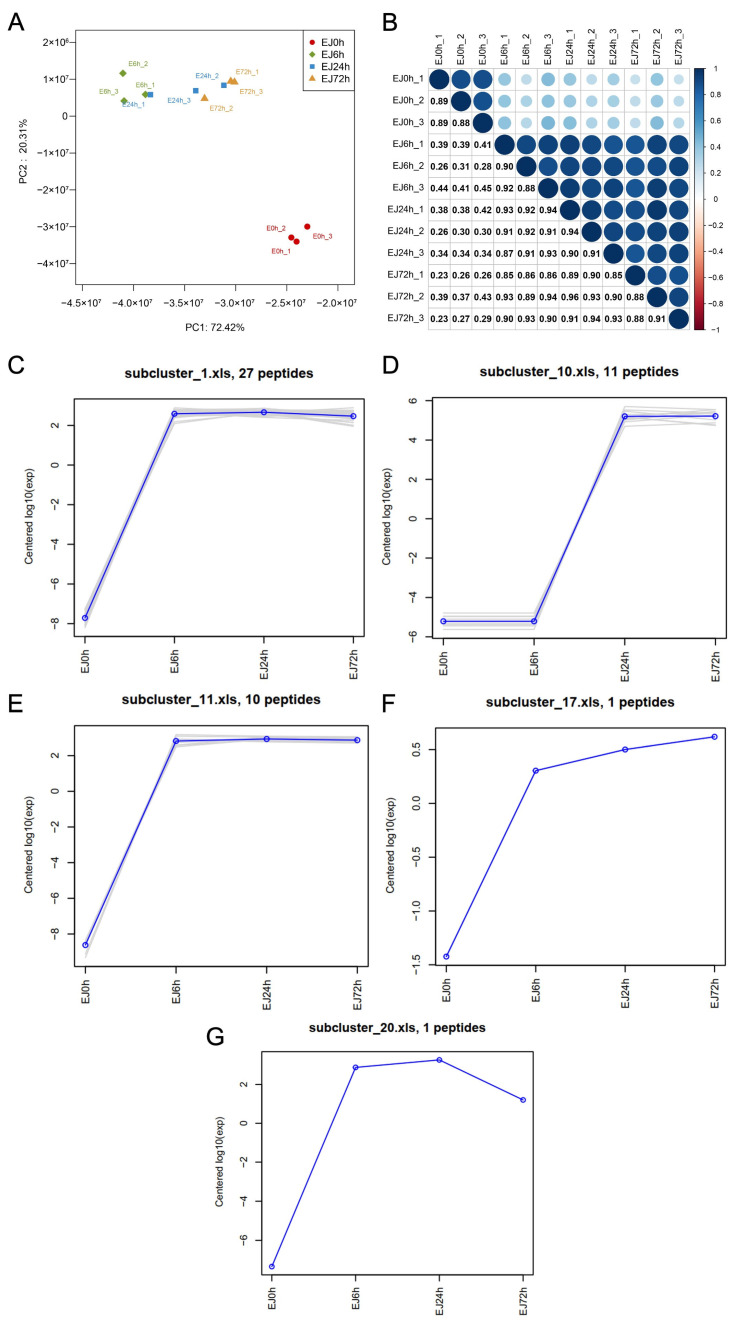
Dynamic changes in the peptidome during Ejiao fermentation by *L. salivarius* D3-8. The fermentation experiments were conducted with three biological replicates (*n* = 3 per group). PCA of peptide profiles across different time points (**A**). Hierarchical clustering analysis of peptides, highlighting distinct expression patterns over time (**B**). Clustering analysis of peptide abundance (**C**–**G**). In panels (**C**–**E**), the blue line represents the mean of all measurements, and the gray lines represent the measured values.

To directly validate the proteolytic activity of *L. salivarius* D3-8 predicted by genomic analysis, we performed a time-course peptidomic analysis of Ejiao medium before and after fermentation. PCA and hierarchical clustering analysis of the peptide profiles revealed a clear and progressive separation over time (Figure 3A,B). The peptide composition at 6, 24, and 72 h post-fermentation was significantly distinct from that at the 0-h time point, demonstrating a substantial remodeling of the Ejiao peptidome by *L. salivarius* D3-8 (Figure 3A,B). While bioactive peptides from Ejiao have previously been generated via in vitro enzymatic digestion or simulated gastrointestinal hydrolysis [13,31], our study provides the first direct evidence that a human gut bacterium can ferment and degrade intact Ejiao into a distinct peptide profile.

To characterize the novel peptides generated, we conducted an in-depth analysis of the fermentation-specific peptidome. Clustering analysis classified the newly formed peptides into 20 distinct subclusters based on their sequence features and abundance patterns. Notably, we identified 50 unique peptides that were exclusively present in the fermented samples but entirely absent in the sterile Ejiao control (Figure 3C–G, Table 4). These peptides represent direct cleavage products of Ejiao proteins by the bacterial proteases. Their sequences provide crucial insights into the potential cleavage sites and specificity of the proteolytic system encoded by *L. salivarius* D3-8. The generation of this specific peptide repertoire not only confirms the bacterium’s role as an active Ejiao degrader but also suggests a potential mechanism by which microbial metabolism transforms a complex macromolecular medicine into a suite of potentially bioactive fragments that may mediate the observed therapeutic effects.

### 3.4. Administration of L. salivarius D3-8 Alone Dose-Dependently Ameliorates DSS-Induced Colitis

Having established that *L. salivarius* D3-8 can degrade Ejiao in vitro, we next sought to determine whether this bacterium, by itself, could confer therapeutic benefits in vivo, independent of the intact Ejiao complex. To this end, we evaluated the efficacy of live *L. salivarius* D3-8 administration in a murine model of DSS-induced colitis. Mice were randomly assigned to receive either a high (LSH), medium (LSM), or low (LSL) dose of the bacterium via daily oral gavage over an eight-day period concurrent with DSS exposure.

**Figure 4:**
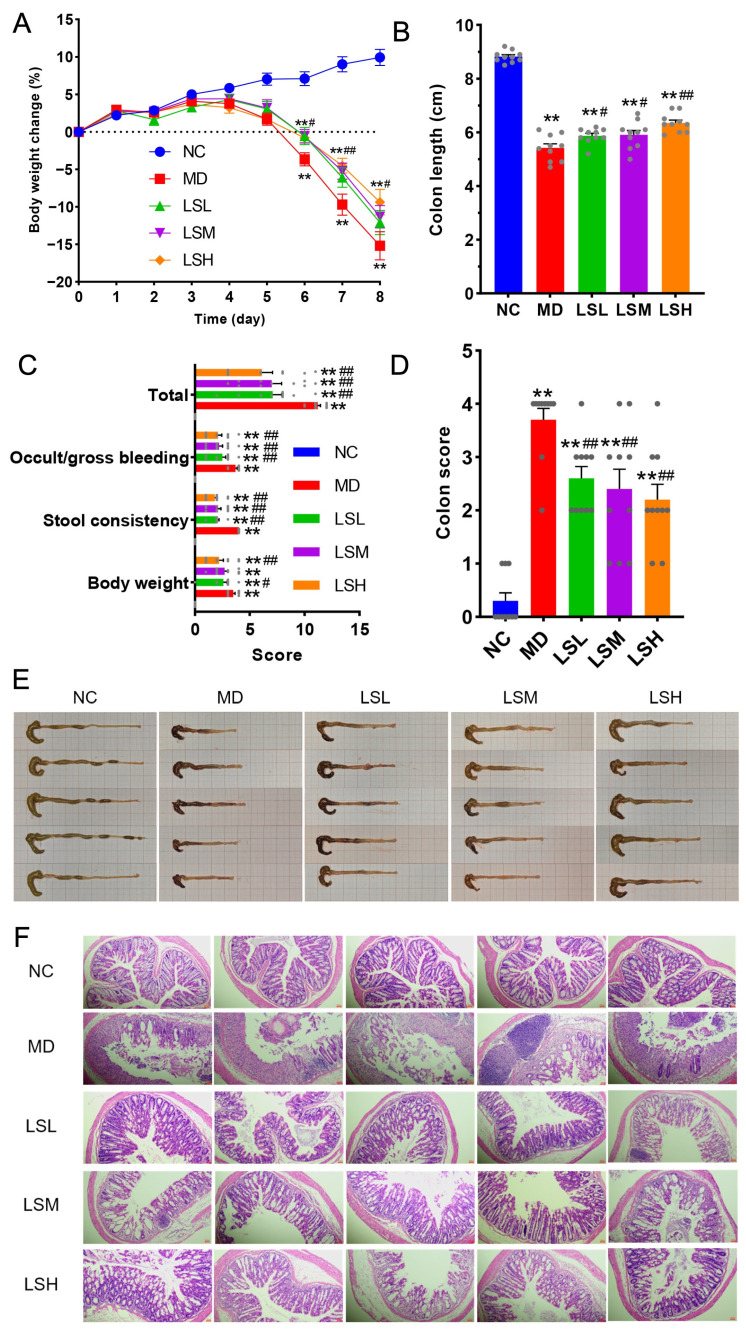
Treatment with *L. salivarius* D3-8 significantly ameliorated DSS-induced colitis (*n* = 10 per group). Changes in body weight during the experimental period (**A**). Colon length at the endpoint (**B**). Disease activity index (DAI) scores (**C**). Histopathological scores of the colon tissues (**D**). Representative macroscopic images of colons from each group (**E**). Representative H&E-stained colon sections (scale bar, 100 μm) (**F**). ** *p* < 0.01 versus NC group; # *p* < 0.05 versus MD group; ## *p* < 0.01 versus MD group. The grey dots in panels (**B**–**D**) represent the experimental values. Different colors in panels (**A**–**D**) represent different experimental groups.

Treatment with *L. salivarius* D3-8 significantly alleviated the hallmark symptoms of colitis in a dose-dependent manner (Figure 4). Mice in the high-dose (LSH) group exhibited a markedly attenuated loss of body weight compared to the DSS-only control group, whereas the protective effect was less pronounced and statistically non-significant in the medium- and low-dose groups (Figure 4A–D). This clear dose–response relationship underscores the therapeutic potential of this specific strain. Beyond weight maintenance, administration of *L. salivarius* D3-8 effectively mitigated DSS-induced colonic pathology (Figure 4E,F). Specifically, it significantly ameliorated colon shortening—a key macroscopic indicator of inflammation and tissue damage (Figure 4B,E). Furthermore, the treatment reduced the incidence and severity of rectal bleeding and, as evidenced by histopathological analysis, conferred notable protection against mucosal ulceration, epithelial destruction, and inflammatory cell infiltration (Figure 4F). Collectively, these results demonstrate that *L. salivarius* D3-8 alone is sufficient to recapitulate key protective effects against experimental colitis, positioning it as a functionally potent probiotic candidate whose activity may underpin part of Ejiao’s known efficacy.

### 3.5. L. salivarius D3-8 Attenuated Gut Dysbiosis by Enriching the Probiotic Dubosiella newyorkensis

Given the established pivotal role of gut microbiota in both the pathogenesis and treatment of ulcerative colitis, we investigated whether the protective effects of *L. salivarius* D3-8 were associated with modulation of the microbial community. We performed 16S rRNA gene sequencing on fecal samples from the normal control (NC), DSS-induced model (MD), and high-dose *L. salivarius* D3-8 treatment (LSH) groups.

**Figure 5:**
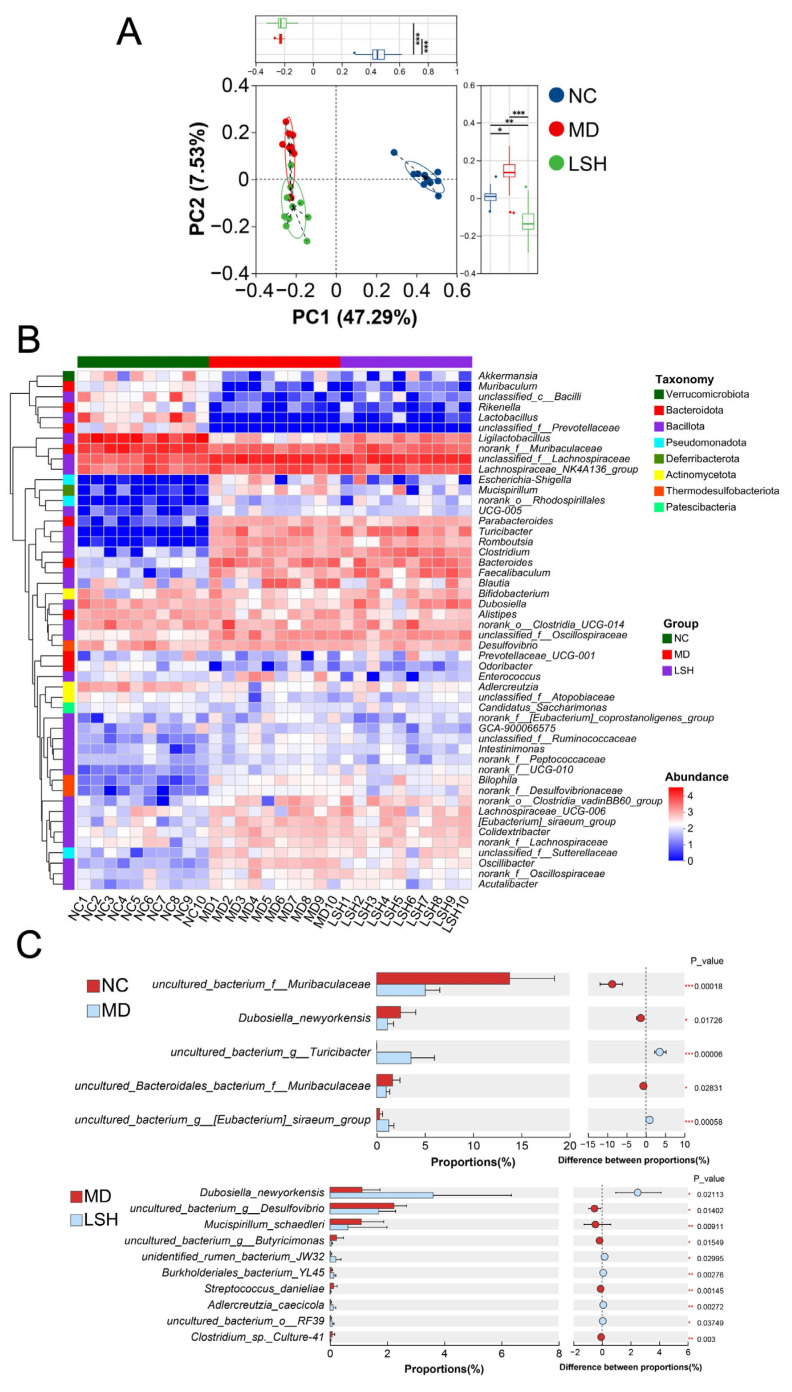
Modulation of the gut microbiota by *L. salivarius* D3-8 in DSS-induced colitis (*n* = 10 per group). PCA of bacterial community structure based on Bray–Curtis distances (**A**). Heatmap illustrating the relative abundance of major bacterial taxa at the genus level (**B**). Significantly altered genera identified by Wilcoxon rank-sum test (**C**). * *p* < 0.05; ** *p* < 0.01; *** *p* < 0.001.

PCA revealed a distinct separation of the overall microbial community structure among the three groups, indicating that both DSS induction and subsequent bacterial treatment significantly reshaped the gut microbiota (Figure 5A). A heatmap visualizing the relative abundance of major genera further illustrated these compositional shifts (Figure 5B). To identify specific bacterial taxa altered by *L. salivarius* D3-8 administration, we performed Wilcoxon rank-sum tests. This analysis identified several genera whose abundance was significantly modulated in the LSH group compared to the MD group (Figure 5C). Most notably, the treatment selectively and significantly increased the abundance of *Dubosiella newyorkensis* (Figure 5C). *D. newyorkensis* is a robust SCFA-producing commensal bacterium previously reported as a potential probiotic capable of ameliorating colitis [32,33,34]. The enrichment of this beneficial commensal suggests a possible mechanism through which *L. salivarius* D3-8 exerts its therapeutic effect, potentially by fostering a microbiota environment conducive to mucosal healing and anti-inflammatory responses.

### 3.6. L. salivarius D3-8 Ameliorates Colitis by Upregulating Tryptophan Metabolism and Elevating the Anti-Inflammatory Metabolite Indole-3-Carbinol

Building on the observed remodeling of the gut microbiota, we hypothesized that these compositional shifts would translate into functional changes in the microbial metabolome, which could underpin the therapeutic effects. To test this, we performed a non-targeted metabolomic analysis on fecal samples from the NC, MD, and LSH groups.

**Figure 6:**
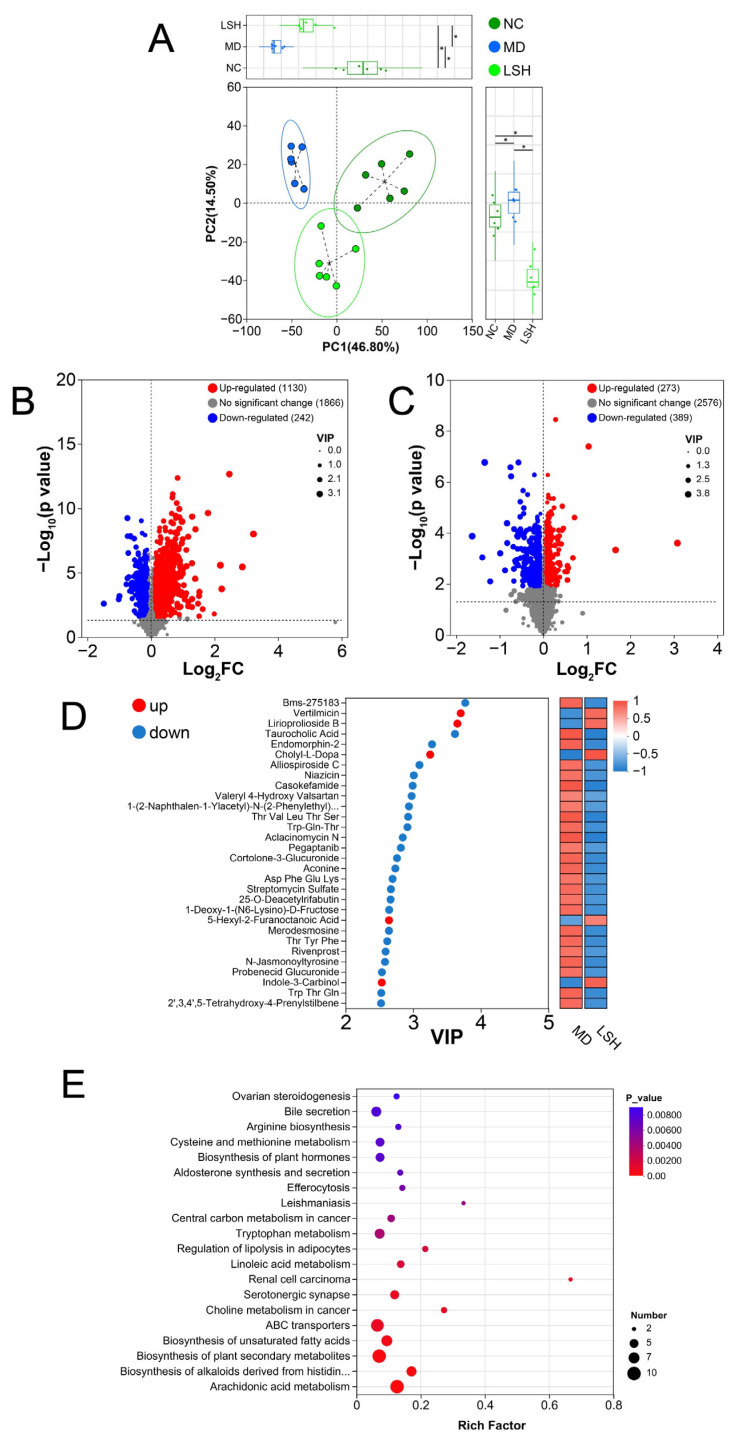
*L. salivarius* D3-8 modulated the composition of intestinal metabolites in DSS-induced C57BL/6J mice by increasing the concentrations of anti-inflammatory inole-3-carbinol (*n* = 6 per group). PCA of the metabolites (**A**). Volcano plot of the metabolites in the MD group vs. NC group (**B**). Volcano plot of the metabolites in the LSH group vs. MD group (**C**). VIP analysis of the metabolites in the LSH group vs. MD group (**D**). KEGG pathway enrichment analysis of the metabolites in the LSH group vs. MD group (**E**). * *p* < 0.05.

PCA of the metabolic profiles demonstrated a clear separation among the three groups, indicating that both colitis induction and *L. salivarius* D3-8 treatment significantly altered the intestinal metabolic landscape (Figure 6A). To identify specific metabolites responsible for this shift, we conducted a Volcano plot analysis comparing the MD versus NC groups, which highlighted the metabolic disturbances caused by DSS (Figure 6B). More importantly, comparison between the LSH and MD groups revealed metabolites that were significantly restored or modulated by bacterial treatment (Figure 6C). From this analysis, VIP scoring identified indole-3-carbinol (I3C) as one of the most significantly elevated metabolites in the LSH group compared to the MD group (Figure 6D). Notably, I3C is a well-documented anti-inflammatory derivative of glucobrassicin with reported efficacy in UC models [35,36].

To understand the metabolic pathway leading to I3C enrichment, we performed KEGG pathway enrichment analysis. This revealed that tryptophan metabolism was a significantly upregulated pathway following *L. salivarius* D3-8 administration (Figure 6E). This finding is mechanistically coherent, as the synthesis of I3C is intrinsically linked to host and microbial processing of tryptophan [37]. Therefore, our data establish a functional chain of evidence: *L. salivarius* D3-8, by modulating the gut microbial community, enhances microbial tryptophan metabolic activity. This, in turn, leads to an increased production of the beneficial metabolite I3C, providing a plausible biochemical mechanism for the anti-colitis effects of this Ejiao-degrading bacterium.

## 4. Discussion

**Figure 7:**
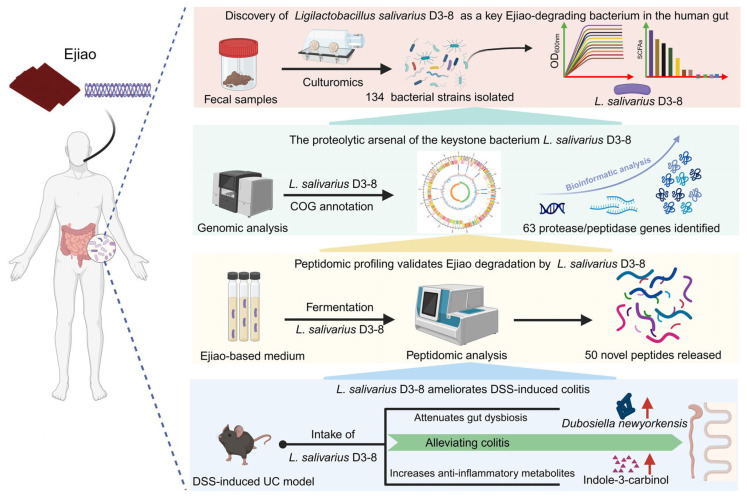
Schematic illustration of the key findings of our study. The figure was created with BioRender.com (accessed on 10 December 2025).

Our study provides a direct link between *L. salivarius* D3-8, a specific bacterium in the human gut, and the therapeutic efficacy of the macromolecular Ejiao against UC (Figure 7). *L. salivarius* D3-8 was not only the most efficient degrader of Ejiao in vitro but also, when administered alone, sufficiently ameliorated DSS-induced colitis in mice. Our finding offers new evidence that the biological effects of Ejiao may be mediated through its interaction with a dedicated bacterial degrader. This could, to some extent, help to resolve the central paradox of how a poorly absorbable macromolecule exerts its pharmacological action.

This work goes beyond viewing gut bacteria as passive consumers of dietary or medicinal compounds [38,39]. We tentatively propose that *L. salivarius* D3-8 might act both as a “keystone degrader” and a “functional effector bacterium” for Ejiao. Its genomic arsenal of proteases and peptidases enables the primary breakdown of the complex collagen matrix in the gut. In this regard, this strain may initiate the transformation of Ejiao from an inert macromolecule into a suite of potentially bioactive peptides. Thus, the efficacy of Ejiao may be conceptualized as the result of a targeted symbiotic partnership, where the medicine provides a selective nutrient for a functionally potent bacterium, which in turn executes protective functions.

The mechanism by which *L. salivarius* D3-8 alleviates colitis appears to be multifaceted. First, the strain modulated the structure of the intestinal community, significantly enriching for the beneficial bacterium *D. newyorkensis*. Second, *L. salivarius* D3-8 upregulated the microbial tryptophan metabolism, leading to an increased level of the anti-inflammatory metabolite I3C. Altogether, these results connect a specific bacterial function to a host-relevant immunomodulatory output, providing a plausible biochemical bridge between bacterial metabolism and host anti-inflammatory response.

Our findings lead us to propose a potential novel mechanistic framework—termed the “Bacterial Degrader-Functional Mediator” paradigm—to explain the pharmacology of macromolecular TCMs such as Ejiao. In this model, the therapeutic substance acts not as a direct ligand for host receptors, but as a selective substrate for key gut bacteria. The identity and functional potency of these microbial degraders determine the metabolic fate of the TCM and its subsequent biological outcomes, thereby shifting the pharmacological focus from the drug *per se* to the microbial catalysts that unlock its bioactivity. This framework has potential implications for the rational development of both TCMs and probiotics. It advocates for the proactive screening and incorporation of defined, functionally validated bacterial strains (exemplified by *L. salivarius* D3-8) as integral components of next-generation TCM formulations, paving the way for more consistent, potent, and mechanism-based therapies.

The therapeutic potential of traditional Chinese medicine in UC management has been increasingly recognized, with accumulating evidence highlighting the critical role of gut microbiota as a mediator of TCM efficacy [40]. A comprehensive review by Zhang et al. systematically summarized how various Chinese herbal medicines and formulas exert anti-inflammatory effects through modulation of both gut microbiota composition and key signaling pathways, including TLR4, NF-κB, STAT3, and PI3K/Akt [40]. Our findings extend this body of knowledge by providing a concrete example of a specific bacterium—*L. salivarius* D3-8—that directly metabolizes a TCM component (Ejiao) and independently confers protection against experimental colitis.

The potential clinical translatability of our findings is further supported by recent real-world evidence demonstrating that multi-component nutraceutical supplementation can enhance conventional therapy outcomes in UC patients [41]. Tursi et al. reported that adding an *Hericium erinaceus*-based multi-compound to 5-ASA therapy significantly improved clinical remission rates and reduced fecal calprotectin levels compared to 5-ASA alone, without adverse events [41]. Although our study is preclinical, it similarly raises the possibility that specific natural products or their microbial metabolites—such as the novel peptides generated by *L. salivarius* D3-8-mediated Ejiao degradation—could serve as effective adjuncts to standard treatments. Moreover, the observation that *L. salivarius* D3-8 alone ameliorates colitis suggests that probiotic strains capable of metabolizing TCM components may represent a novel class of “TCM-biotic” therapeutics worthy of further investigation.

Through culturomics-based screening of the human gut microbiota, we isolated 134 bacterial strains capable of utilizing Ejiao as a sole nutrient source, underscoring the substantial capacity of the gut microbial community to metabolize this macromolecular traditional medicine. Among these isolates, *L. salivarius* D3-8 demonstrated the most robust growth in Ejiao-based medium and was therefore selected as the primary model organism for investigating the functional consequences of Ejiao degradation. However, it is important to acknowledge that the remaining 133 isolates—while not characterized in detail in the present study—may also possess the ability to process Ejiao components and could potentially contribute to its biological effects. Indeed, the therapeutic efficacy of Ejiao in vivo likely reflects the collective activity of multiple bacterial degraders within a complex microbial ecosystem, rather than the action of a single strain in isolation. Future studies employing metagenomic and metabolomic approaches in gnotobiotic animals colonized with defined consortia of Ejiao-utilizing bacteria will be necessary to dissect potential synergistic interactions and to determine whether different degraders generate distinct bioactive products. Nevertheless, the identification of *L. salivarius* D3-8 as a potent Ejiao degrader with intrinsic anti-colitis properties provides a foundational platform for understanding how specific microbial members may interface with traditional medicines to influence host health.

Several questions remain for future investigation. First, the specific bioactive peptides released by *L. salivarius* D3-8 require functional validation. Second, the ecological interactions between *L. salivarius* D3-8 and *D. newyorkensis*, and whether their co-administration yields synergistic effects, warrant further exploration. Third, verifying the presence and activity of *L. salivarius* D3-8 or analogous degraders in human patients responding to Ejiao therapy will be crucial for clinical translation.

We acknowledge several limitations in the present study. First, while we have demonstrated that *L. salivarius* D3-8 can degrade Ejiao in vitro and that administration of D3-8 alone ameliorates DSS-induced colitis in vivo, our experimental design does not establish a definite causal relationship between these two observations. Specifically, we lack formal mediation experiments to determine whether the protective effects of Ejiao are dependent on the presence or metabolic activity of *L. salivarius* D3-8. Second, we did not directly assess the colonization efficiency or intestinal persistence of exogenously administered *L. salivarius* D3-8, which precludes definitive conclusions regarding its in situ metabolic contribution to Ejiao processing. Third, the observed increase in *D. newyorkensis* abundance following *L. salivarius* D3-8 administration, while intriguing, remains correlative; whether this represents a direct syntrophic interaction, a secondary consequence of altered gut ecology, or a phenomenon independent of *L. salivarius* D3-8 activity requires validation through co-culture experiments and targeted depletion studies. Fourth, although metabolomic profiling revealed elevated I3C levels and enhanced tryptophan metabolism, we did not perform functional blockade experiments (e.g., pharmacological inhibition or genetic knockout of key metabolic enzymes) to confirm that this pathway directly mediates the anti-inflammatory effects observed. Consequently, our proposed mechanistic model—wherein *L. salivarius* D3-8 degrades Ejiao, promotes *D. newyorkensis* expansion, and modulates tryptophan metabolism to alleviate colitis—should be interpreted as a hypothesis-generating framework rather than a definitively validated pathway. Future studies employing gnotobiotic animal models, bacterial depletion strategies, and targeted metabolic interventions will be necessary to establish causality and elucidate the precise molecular mechanisms underlying the complex interactions among Ejiao, *L. salivarius* D3-8, and the gut microbiota.

We also note that our in vivo experiments did not include established positive controls such as mesalazine or clinically validated probiotic strains. While the inclusion of such comparators would facilitate quantification of relative therapeutic efficacy and provide a clinical benchmark, the primary goal of the present study was to establish a proof-of-concept that an Ejiao-degrading bacterium could independently confer protection against colitis. Future studies incorporating standard-of-care controls will be necessary to rigorously quantify the comparative efficacy of *L. salivarius* D3-8 and to assess its potential translational value.

## 5. Conclusions

In this study, we demonstrate that *L. salivarius* D3-8, a human gut bacterium isolated through culturomics-based screening, functions both as a dedicated degrader of Ejiao and a protective probiotic against experimental colitis. Genomic and peptidomic analyses confirmed its proteolytic capacity, leading to the generation of novel peptides from Ejiao. Importantly, administration of *L. salivarius* D3-8 alone ameliorated DSS-induced colitis, accompanied by enhanced abundance of the beneficial bacterium *D. newyorkensis* and elevated levels of the anti-inflammatory metabolite I3C via modulation of tryptophan metabolism. These findings demonstrate that specific gut bacteria can metabolize macromolecular components of traditional medicines such as Ejiao, raising the possibility that this interaction might play a role in mediating their therapeutic efficacy. Our work establishes a novel mechanistic paradigm in which the biological activity of poorly absorbable traditional drugs may depend on functional interactions with gut microbial degraders. Furthermore, *L. salivarius* D3-8 represents a promising probiotic candidate for the management of ulcerative colitis and highlights the potential of integrating culturomics, multi-omics, and functional validation to decode microbiota–drug interactions.

## Figures and Tables

**Table 1:** Summary of Ejiao-degrading bacterial strains isolated from human feces.

Obtained Ejiao-Degrading Bacteria	Number of Strains
*Bifidobacterium longum* subsp. *longum*	21
*Bifidobacterium longum* subsp. *infantis*	2
*Bifidobacterium faecale*	18
*Bifidobacterium pseudocatenulatum*	20
*Limosilactobacillus fermentum*	24
*Liquorilactobacillus nagelii*	1
*Lactococcus lactis* subsp. *lactis*	2
*Enterococcus durans*	30
*Shigella dysenteriae*	1
*Ligilactobacillus salivarius*	13
*Limosilactobacillus mucosae*	2

**Table 2:** Summary of protease-related genes identified in the genome of *L. salivarius* D3-8.

Gene ID	Location	COG Description
gene0059; gene1111	Chromosome	Periplasmic serine protease, S1-C subfamily, contains C-terminal PDZ domain
gene0073; gene0214; gene0342; gene0795	Chromosome	ATP-dependent Clp protease, ATP-binding subunit ClpA
gene0135; gene1123; gene1489; gene1629	Chromosome	Membrane protease YdiL, CAAX protease family
gene0087	Chromosome	Predicted Zn-dependent protease
pA gene0044	Plasmid A	Membrane protease YdiL, CAAX protease family
gene0236	Chromosome	Zn-dependent protease with chaperone function
gene0265	Chromosome	Serine protease, subtilase family
gene0547	Chromosome	Membrane-associated serine protease, rhomboid family
gene0563	Chromosome	Membrane-associated protease RseP, regulator of RpoE activity
gene0642	Chromosome	ATP-dependent protease Clp, ATPase subunit ClpX
gene0783	Chromosome	Outer membrane protein chaperone/metalloprotease BepA/YfgC, contains M48 and TPR domains
gene0874	Chromosome	ATP-dependent protease HslVU (ClpYQ), ATPase subunit HslU
gene0875	Chromosome	ATP-dependent protease HslVU (ClpYQ), peptidase subunit
gene1096	Chromosome	ATP-dependent protease ClpP, protease subunit
gene1169	Chromosome	Predicted Zn-dependent metalloprotease, SprT family
gene1283	Chromosome	ATP-dependent Zn proteases
pA gene0119	Plasmid A	Serine protease, subtilisin family
pA gene0121	Plasmid A	Regulator of protease activity HflC, stomatin/prohibitin superfamily

**Table 3:** Summary of peptidase-related genes identified in the genome of *L. salivarius* D3-8.

Gene ID	Location	COG Description
gene0012; gene1459	Chromosome	Murein DD-endopeptidase MepM and murein hydrolase activator NlpD, contains LysM domain
gene0037; gene0371	Chromosome	Oligoendopeptidase F
gene0044; gene0130; gene0311; gene0179	Chromosome	Lipoprotein-anchoring transpeptidase ErfK/SrfK
gene0093; gene0309; gene1445	Chromosome	D-alanyl-D-alanine carboxypeptidase
gene0182; gene0932	Chromosome	Dipeptidase
gene0217	Chromosome	Prepilin signal peptidase PulO (type II secretory pathway) or related peptidase
gene0388; gene1007	Chromosome	Xaa-Pro aminopeptidase
gene0469	Chromosome	Metal-dependent amidase/aminoacylase/carboxypeptidase
gene0472; gene0865	Chromosome	Penicillin-binding protein 1B/1F, peptidoglycan transglycosylase/transpeptidase
gene0538	Chromosome	SOS-response transcriptional repressor LexA (RecA-mediated autopeptidase)
pA gene0170	Plasmid A	SOS-response transcriptional repressor LexA (RecA-mediated autopeptidase)
gene0583	Chromosome	CubicO group peptidase, beta-lactamase class C family
gene0756	Chromosome	Lipoprotein signal peptidase
gene0780	Chromosome	Di- or tripeptidase
gene0808	Chromosome	Signal peptidase I
gene0875	Chromosome	ATP-dependent protease HslVU (ClpYQ), peptidase subunit
gene0976; gene1012	Chromosome	Cell division protein FtsI, peptidoglycan transpeptidase (Penicillin-binding protein 2)
gene1060; gene1061	Chromosome	Predicted Zn-dependent peptidase, M16 family
gene1163	Chromosome	Aminopeptidase C
gene1187	Chromosome	Methionine aminopeptidase
gene1195	Chromosome	Leucyl aminopeptidase (aminopeptidase T)
gene1302	Chromosome	D-alanyl-D-alanine dipeptidase
gene1448	Chromosome	Predicted cysteine peptidase, C39 family
gene1519	Chromosome	Sortase (surface protein transpeptidase)
gene1569	Chromosome	Predicted metalloendopeptidase
pA gene0045	Plasmid A	Aminopeptidase N, contains DUF3458 domain

**Table 4:** Identification and sequence features of the 50 novel peptides generated during Ejiao fermentation by *L. salivarius* D3-8 (72 h vs. 0 h).

Subcluster	Accession	Annotated Sequence
1	peptide 467	VVGKPGIPTGPI
	peptide 461	VMGPAGSRGATGPA
	peptide 399	SIVGRPR
	peptide 85	DIVPGDIVEV
	peptide 313	PIQSPLPVIPH
	peptide 460	VLDRPGPPEGPL
	peptide 269	LFDKPVSPL
	peptide 476	YELPDGQV
	peptide 308	PHQYPALTPEQ
	peptide 19	AGPAGPAGPIGPVGARGPA
	peptide 154	GEAGPAGPAGPIGPVGARGPAGPQ
	peptide 60	DAPRAVFPSIVGRPRHQ
	peptide 75	DGSVGPVGPAGPI
	peptide 442	VFPSIVGRPR
	peptide 117	DVPGPPTGPI
	peptide 116	DVPGPPGPIEI
	peptide 420	SVGPVGPAGPI
	peptide 46	APPIQSPLPVIPH
	peptide 211	GPMGLMGPR
	peptide 452	VGPVGPAGPI
	peptide 100	DRGEAGPAGPAGPIGPVGAR
	peptide 133	FPSIVGRP
	peptide 238	GRPGPPVGPI
	peptide 239	GSDGSVGPVGPAGPI
	peptide 27	AGPAGPIGPV
	peptide 240	GSVGPVGPAGPI
	peptide 307	PGPMGPSGPR
10	peptide 195	GLDGLPGVPG
	peptide 25	AGPAGPAGPIGPVGARGPAGPQGPRG
	peptide 205	GPAGPAGPIGPV
	peptide 23	AGPAGPAGPIGPVGARGPAGPQG
	peptide 132	FLPQPPQE
	peptide 153	GEAGPAGPAGPIGPVGARGPAG
	peptide 425	SVWIGGSI
	peptide 303	PGPMGLM(+15.99)GPRGPPGA
	peptide 248	GVPGPPGAVGPA
	peptide 209	GPIGPVGARGPAGPQ
	peptide 310	PIERPSPPV
11	peptide 52	AVFPSIVGRPR
	peptide 51	AVFPSIVGRP
	peptide 134	FPSIVGRPR
	peptide 216	GPVGPTGPV
	peptide 208	GPAGPIGPV
	peptide 441	VFPSIVGRP
	peptide 260	IKIIAPPE
	peptide 127	FDKPVSPL
	peptide 329	PSIVGRPR
	peptide 298	PGEPGPQGPIGVP
17	peptide 190	GISVPGPMG
20	peptide 246	GVGVLPGVPT

## Data Availability

The whole genome sequence of *L. salivarius* D3-8 was deposited in GenBank under the accession number CM133239.1 and JBSWZN010000002.1. The BioProject and BioSample accession numbers were PRJNA1368413 and SAMN53363752, respectively.

## Abbreviations

The following abbreviations are used in this manuscript:

SCFAs	Short-chain fatty acids
COG	Clusters of orthologous groups
OD	Optical density
DSS	Dextran sulfate sodium
LC-MS	Liquid chromatography coupled with tandem mass spectrometry
PCA	Principal component analysis
SPF	Specific-pathogen-free
UC	Ulcerative colitis
VIP	Variable importance in projection
KEGG	Kyoto Encyclopedia of Genes and Genomes
I3C	Inole-3-carbinol
TCM	Traditional Chinese medicine
